# The quality of anti-malarial medicines in Embu County, Kenya

**DOI:** 10.1186/s12936-018-2482-3

**Published:** 2018-09-15

**Authors:** Stanley Ndwigah, Andy Stergachis, Kennedy Abuga, Hannington Mugo, Isaac Kibwage

**Affiliations:** 1 Department of Pharmaceutical Chemistry, School of Pharmacy, University of Nairobi, P.O. Box 19676-00202, Nairobi, Kenya; 2 Department of Pharmacy, School of Pharmacy, University of Washington, P.O. Box 357236, Seattle, WA 98105 USA; 3 Department of Global Health, School of Public Health, University of Washington, P.O. Box 357236, Seattle, WA 98105 USA

**Keywords:** Quality, Anti-malarial drugs, Artemisinin-based combination therapy, Monotherapy, Kenya

## Abstract

**Background:**

Malaria is a major health problem in sub-Saharan Africa where over 90% of the world’s malaria cases occur. Artemisinin-based combination therapy (ACT) is recommended by the World Health Organization as first-line and second-line treatments for uncomplicated falciparum malaria. However, there are a growing number of reports of sub-standard and falsified anti-malarial medicines in sub-Saharan Africa.

**Methods:**

A cross-sectional study was conducted in Embu County, Kenya on the quality of anti-malarial medicines available in public and private facilities. Sampling of anti-malarial medicines from public and private hospitals, health centers and pharmacies was conducted between May and June 2014. Quality control tests were performed at the Drug Analysis and Research Unit, University of Nairobi, using ultraviolet spectrophotometry and high-performance liquid chromatography. A test for microbial load was also conducted for suspension formulations.

**Results:**

A total of 39 samples were collected from public and private facilities across the Embu County. A visual inspection of the medicines showed no signs of sub-standard or falsification. All ACT passed identification, assay and dissolution tests. Of 11 suspension samples collected, none failed the microbial load test although one sample had 50 colony forming units (cfu). No oral artemisinin monotherapy medicines were encountered during the survey. Amodiaquine and chloroquine monotherapy products accounted for 5% of the collected samples, despite their ban in Kenya. Two herbal anti-malarial formulations were collected during the survey. Sulfadoxine/pyrimethamine (SP) was also found to be available use for malaria treatment, not in accordance with malaria treatment guidelines.

**Conclusion:**

All the anti-malarial drugs analysed in this study passed the quality control tests. This is encouraging given the high malaria burden in Kenya. Regulatory actions are required to counter SP and herbal products for malaria treatment.

**Electronic supplementary material:**

The online version of this article (10.1186/s12936-018-2482-3) contains supplementary material, which is available to authorized users.

## Background

An estimated 3.3 billion people live in areas at risk of malaria transmission, with those living in sub-Saharan Africa at the highest risk. Malaria is a preventable and treatable disease when recommended interventions are properly implemented. These include chemoprophylaxis for pregnant women and timely treatment with appropriate anti-malarial medicines [1]. A vital component of malaria control depends on the availability of quality-assured artemisinin-based combination therapy (ACT) provided in correct dosages. However, there are increasing reports of sub-standard anti-malarials circulating in sub-Saharan Africa [2–8]. There is also a reported increase of falsification, including counterfeiting of anti-malarial pharmaceuticals partly due to the rise of the illegal, informal market and to the relatively high cost of branded products [9]. The availability of non-recommended, over-the-counter medicines for malaria treatment and the presence of sub-standard anti-malarials in the market have been documented in Kenya [10]. Studies carried out elsewhere on the quality of anti-malarials in circulation have shown concerning levels of sub-standard and counterfeit medicines [11, 12].

The presence of artemisinin monotherapy, sub-standard and fake medicines containing sub-therapeutic amounts of anti-malarials increase the risk of recrudescence of malaria infections and promotes the development of drug resistance [12]. Knowledge about the actual quality of medicines available within a given community is important for formulation of suitable policies, enforcement activities and other practices aimed at improving malaria treatment [7]. The Ministry of Health, Pharmacy and Poison’s Board (PPB), civil society groups, and others concerned about quality of medicines are encouraged to undertake post-marketing quality surveillance as having access to reliable and valid data is an important initial step to determining policy options and actions to improve the availability and affordability of quality-assured medicines. According to the United States Pharmacopoeia (USP) and World Health Organization (WHO) [13, 14], the minimal tests required to evaluate the quality of medicinal products are that a sample must pass one test before moving on to the next test. These tests include identity, assay, disintegration, and dissolution. The objective of this study was to determine the quality of anti-malarial medicines available at health facilities within Embu County, Kenya. The anti-malarial drugs were tested for identity, assay, dissolution, and microbial load.

## Methods

### Setting

This study was conducted in Embu County, Kenya, from May to June 2014. The total population of Embu County is 516,212 [15] with Embu town as the county headquarters. Embu County is administratively divided into 5 sub-counties, each served by a public hospital plus several health centres and dispensaries.

A total of 6 hospitals, 13 health centres and 29 private pharmacies were recruited for the study. Facilities visited were 5 public hospitals as well as 6 public health centres spread across the sub-counties. In addition, one mission hospital, 2 mission health centres, 5 private-for-profit health facilities and 29 private pharmacies were also visited. The private pharmacies sampled were proximal to the health facilities and were considered to be the alternative sources of medications in case of stock-outs at the public facilities.

The medical supplies distribution chain in Kenya is under the statutory regulation of the Pharmacy and Poisons Board (PPB) for both the public and private sectors. The private sector facilities obtain their supplies from commercial distributers and wholesalers who are licensed for that purpose whereas public health facilities are served by a central procurement agency, the Kenya Medical Supplies Agency (KEMSA) which operates as a state corporation. Faith/mission-based institutions operate a central supplies agency, the Mission for Essential Drugs and Supplies (MEDS) which supplies medicines for these facilities. Both private and public facilities stocked first-line AL products under the Affordable Medicines Facility-malaria (AMfm) subsidy programme.

### Sampling method

The study team interviewed the superintendent pharmacists or pharmaceutical technologists of the private pharmacies and medical superintendent and the pharmacist-in-charge or pharmaceutical technologist-in-charge of the hospitals. Through these interviews, information was obtained about which anti-malarials were stocked at their facilities.

Sampling of anti-malarials was performed by the study team during field visits. Sampling was performed according to the active pharmaceutical ingredient (API) profile. For purposes of this study, a sample was defined as a medicine with a given API, dosage form, strength, and batch number. Samples with the same attributes and the same batch number were collected only once. While the intent was to only sample drugs recommended for malaria case treatment by WHO [16], other products were encountered as purported malaria treatments and were included in the quality analysis. These included sulfadoxine/pyrimethamine (SP) tablets, quinine, amodiaquine and chloroquine suspensions, and herbal anti-malarial formulations. Sufficient amounts of samples were purchased for each available product in order to carry out identification, assay, dissolution and other specific tests.

The drugs were first screened by visual inspection for signs of counterfeiting such as improper packaging, labelling or description of dosage, using a checklist produced by the International Council of Nurses in partnership with the United States Pharmacopoeia (USP) and modified by the Military and Emergency Pharmacists Section of FIP [17, 18].

### Analytical process

Quality control tests were carried out at the Drug Analysis and Research Unit (DARU), University of Nairobi. The DARU is a laboratory in Kenya established in the Department of Pharmaceutical Chemistry that conducts routine analysis on pharmaceutical products as well as post-marketing quality surveillance for government agencies and others. Analysis was carried out using HPLC, UV spectrophotometry and microbial methods, according to procedures as specified by USP [13] and in-house methods. Tablets were subjected to identification, assay and dissolution tests. Liquid dosage forms were analysed for content and microbial load. Certificates of analysis were issued for each sample.

The chromatographic system consisted of HPLC column of dimensions 250 mm × 4.6 mm packed with Rsil C18 5 um, a Shimadzu Prominence LC-20A pump set to deliver 1 ml/min volume of mobile phase, a prominence autosampler model SIL-20A HT, prominence UV–Vis detector model SPD-20A, a Shimadzu column oven model CTO-10ASVP set 40 °C, and an LC solution software. A Genesys 10S UV–Vis spectrophotometer was used to determine absorbance, on portions of the test solutions in comparison with the standard solutions, using 1.0 cm cell and medium as the blank. The dissolution tester used was Erweka model DT6 with a volume of 900 ml medium for all molecules except artemether (1000) ml, Apparatus II (paddle), rpm (100) for artemether/lumefantrine, dihydroartemisinin/piperaquine phosphate tablets and rpm (50) for artesunate/mefloquine and artesunate/amodiaquine tablets and a water bath set at 37 °C.

## Results

A total of 39 different samples were collected from the 48 facilities across Embu County, Kenya. The majority of the samples were obtained from the private sector pharmacies (Table 1). The range of different anti-malarials was highest in the private sector followed by the public sectors.

**Table 1:** Sampling by sector

Sector	Number of samples	Percentage %
Private pharmacies	29	74.4
Public hospitals	6	15.4
Public health centres	4	10.3
Total	39	100

### Visual inspection

The results of visual inspection showed no signs of sub-standard drugs or falsification. All of the anti-malarial products encountered had correct labels about their source manufacturers and all of the labels contained information on strength, dosage and expiration dates.

### Sampling by API

The most frequently sampled medicines were artemether/lumefantrine, quinine and dihydroartemisinin–piperaquine (Table 2).

**Table 2:** Distribution of samples by active pharmaceutical ingredient

Active pharmaceutical ingredient(s)	Total	Percentage
Artemether/lumefantrine	18	46.2
Quinine suspension	6	15.4
Dihydroartemesinin/piperaquine	4	10.3
Artesunate/mefloquine	2	5.1
Unknown—Remoxe^®a^	2	5.1
Amodiaquine	1	2.6
Artemisinin/piperaquine	1	2.6
Artesunate/amodiaquine	1	2.6
Artemisinin/naphthoquine	1	2.6
Chloroquine	1	2.6
Artesunate injection	1	2.6
Artesunate suspension	1	2.6
Total	39	100

^a^Remoxe^®^ is a herbal product made of extracts of *Ajuga remota*. Its use was based on folklore where *Ajuga remota* is used by locals in treatment of malaria. Remoxe^®^ suspension is a white extract of *Ajuga remota* in natural vegetable oil while the capsules are made of *Ajuga remota* powder

### Results from analytical tests

Additional file 1: Table S1 reports the assay and dissolution results of artemether/lumefantrine tablets. Additional file 1: Table S2 shows assay and dissolution results for other ACT medicines. All the artemether/lumefantrine formulations and all of the other ACT medicines complied with identification, assay and dissolution tests as per the pharmacopoeias. Additional file 1: Table S3 shows results of microbial load tests. All of the suspensions collected passed the microbial load test.

## Discussion

The distribution of the sources of samples collected for analysis shows that highest numbers of samples were obtained from the private retail pharmacies followed by the public facilities. In Kenya, the private retail market relies heavily on stocking a wide range of brands of anti-malarials in response to market demand dynamics. Public procurement, on the other hand, is governed by strict adherence to the Kenya essential medicines list (KEML) and rational stocking is based on a minimalistic approach towards brand variety.

Approximately 46% of the samples were artemether–lumefantrine (AL), which is the recommended first-line treatment for malaria in Kenya. This is further supported by the AL subsidy financed by the Global Fund under AMFm model to improve availability and affordability of the medicines. This initiative seems to be achieving its objectives as demonstrated in this study and corroborated by others [19].

Surprisingly, quinine formulations were the second most frequently encountered anti-malarial at 15% with several paediatric products. Clinicians and patients alike may prefer this drug for management of malaria for historical reasons despite the availability of AL in child friendly, dispersible tablets. Quinine suspension is not listed in the EML nor recommended in the Kenyan Malarial Treatment Guidelines [16, 20]. More campaigns are therefore needed to promote use of AL for malaria case management.

Physical and visual examination of the samples collected, including the printed inserts, text on packages, sizes of blister packs and the green ACTm logo where applicable indicated that ACT in Embu County were authentic products. Furthermore, all samples analysed complied with identification test showing the presence of the declared APIs in the respective formulations.

All AL samples complied with the assay and dissolution tests performed, which is an indicator of the quality of the products in the market. Dissolution was not carried on one sample formulated as soft gelatin capsules, for which the test is not prescribed in the compendia. This high compliance rate may be related to the efforts made by the AMFm programme during prequalification of manufacturers for current Good Manufacturing Practices (cGMP) conformance.

Similarly, the other ACT medicines encountered in the market complied with assay and dissolution test. Dihydroartemisinin/piperaquine (DHA/PPQ) co-formulated tablets are the recommended second-line treatment for uncomplicated malaria. Artequick^®^ is presented as a 4-tablet pack for 2-day treatment of uncomplicated malaria. This pack size is at variance with those found in other markets. In Tanzania for instance, the product is sold in a 6-tablet pack to be taken for 3 days. This discrepancy suggests the need for evidence-based rationalization by the manufacturers and the competent authorities within the markets for consistency of dosing across sub-Saharan Africa malaria regions, which have similar infectivity profiles [21]. The other three ACT medicines encountered in this study were artesunate in combination with amodiaquine, mefloquine and naphthoquine with varying dosage regimens.

Only one artesunate injection brand was found in Embu County. When tested, it complied with assay and sterility tests. All of the suspensions complied with the assay test while all but one passed the microbial load limits. The herbal preparation Remoxe^®^ also complied with the microbial load limits test. Amodiaquine and chloroquine monotherapy products were encountered in the market despite their ban in Kenya. Use of these products in malaria cases could endanger the lives of the patients given their documented ineffectiveness.

## Conclusion

All the anti-malarial drugs that were analysed in this study complied with quality control tests. This is encouraging given the high malaria burden in Kenya. Previous reports have reported higher failure rates for anti-malarial drugs in Kenya [22]. Some products containing chloroquine, amodiaquine and herbal components were found in the market not in accordance with malaria treatment guidelines. Regulatory action by PPB is recommended to counter the availability and use of monotherapy and herbal products for malaria treatment.

## Additional file

**Additional file 1: Table S1.** Artemether/lumefantrine assay and dissolution results. **Table S2.** Assay and dissolution results for other artemisinin combination therapy. **Table S3.** Results of microbial load carried out on the liquid and herbal anti-malarials. One quinine suspension had contamination with 50 colony forming units of aerobic bacteria per ml.
