# Supplementary material for: The quality of anti-malarial medicines in Embu County, Kenya
Source: Malar J. 2018 Sep 15;17:330. doi: 10.1186/s12936-018-2482-3 (PMC6138895; doi:10.1186/s12936-018-2482-3)
Supplement: Supplementary file 1 — Additional file 1: Table S1. Artemether/lumefantrine assay and dissolution results. Table S2. Assay and dissolution results for other artemisinin combination therapy. Table S3. Results of microbial load carried out on the liquid and herbal anti-malarials. One quinine suspension had contamination with 50 colony forming units of aerobic bacteria per ml. [file 12936_2018_2482_MOESM1_ESM.docx]

**Table S1. Artemether/lumefantrine assay and dissolution results**

| **DARU No.** | **Brand name** | **Assay (% label claim)** | | **Dissolution (% label claim)** | | |
| --- | --- | --- | --- | --- | --- | --- |
|  |  | **Artemether** | **Lumefantrine** | **Artemether 1 h** | **Artemether 3 h** | **Lumefantrine 45 min** |
| 275/14 | Coartem^®^ dispersible tablets | 102.5 | 98.2 | 107.8 | 109.8 | 80.9 |
| 276/14 | Coartem^®^ dispersible tablets | 109.2 | 101.1 | 105.5 | 107.2 | 80.4 |
| 277/14 | Coartem^®^ dispersible tablets | 101.9 | 99.4 | 107.8 | 107.5 | 72.2 |
| 278/14 | Coartem^®^ dispersible tablets | 100.2 | 98.6 | 101.6 | 106.9 | 75.7 |
| 279/14 | Coartem^®^ dispersible tablets | 103.2 | 96.2 | 101.1 | 101.4 | 93.0 |
| 280/14 | Coartem^®^ dispersible tablets | 102.6 | 99.5 | 98.9 | 100.3 | 97.3 |
| 281/14 | Artemether-Lumefantrine tablets | 103.0 | 105.3 | 93.2 | 95.2 | 85.1 |
| 282/14 | Artemether-Lumefantrine tablets | 98.8 | 100.9 | 110.3 | 111.2 | 89.3 |
| 283/14 | AL-IPCA^®^Actm tablets | 100.4 | 106.0 | 91.9 | 101.3 | 87.0 |
| 284/14 | Artemether Lumefantrine tablets | 97.4 | 92.8 | 104.7 | 108.9 | 85.7 |
| 285/14 | Artemether lumefantrine tablets | 109.4 | 104.6 | 104.0 | 105.0 | 94.3 |
| 286/14 | Co-falcinum^®^ AL Actm | 102.4 | 104.6 | 104.6 | 104.1 | 86.7 |
| 287/14 | Co-falcinum^®^ AL Actm | 103.5 | 106.5 | 114.0 | 114.7 | 86.4 |
| 288/14 | Artefan tablets | 108.3 | 97.7 | 102.2 | 102.1 | 109.6 |
| 289/14 | Lonart DS^®^ tablets | 96.3 | 99.7 | 110.9 | 112.7 | 69.8 |
| 290/14 | Cofantrine Forte^®^ tablets | 106.4 | 104.8 | 100.4 | 99.9 | 31.5 |
| 291/14 | Lumesoft Plus^®^ capsules | 102.5 | 95.9 | No available method of analysis^[[1]](#footnote-1)^ | | |
| 292/14 | Lonart^®^ suspension | 96.5 | 97.8 | n/a | n/a |  |

Lumesoft plus^®^ are soft gelatin capsules

**Table S2 Assay and dissolution results for other artemisinin combination therapy**

| **DARU number** | | **Brand name** | **Assay (% label claim)** | | | **Dissolution (% label claim)** | | |
| --- | --- | --- | --- | --- | --- | --- | --- | --- |
| **1. Dihydroartemisinin/piperaquine** | | | Dihydroartemisinin | Piperaquine | | Dihydroartemisinin | | Piperaquine |
| 293/14 | | Duo-cotexin® tablets | 107.6 | 100.2 | | 97.2 | | 82.5 |
| 294/14 | | P-Alaxin® tablets | 93.9 | 96.9 | | 81.9 | | 81.9 |
| 295/14 | | Ridmal® tablets | 99.4 | 91.2 | | 94.8 | | 89.0 |
| 296/14 | | Darte-Q® capsules | 97.1 | 84.9 | | 89.2 | | 88.3 |
| 297/14 | | Malacur 60 ml | 98.4 | 97.9 | | Not applicable | | |
| **2. Artemisinin/piperaquine** | | | Artemisinin | Piperaquine | | Artemisinin | | Piperaquine |
| 298/14 | | Artequick® tablets | 103.2 | | 98.7 | 104.4 | 90.6 | |
| **3. Artesunate/amodiaquine** | | | Artesunate | | Amodiaquine | Artesunate | Amodiaquine | |
| 300/14 | | Asaq® tablets | 108.7 | | 98.9 | 110.2 | 100.5 | |
| **4. Artesunate/mefloquine** | | | Artesunate | | Mefloquine | Artesunate | Amodiaquine | |
| 302/14 | | Artequin® tablets | 95.2 | | 106.9 | 91.6 | 81.5 | |
| 303/14 | | Artequin®paediatric sachets | 98.4 | | 118.0 | Not applicable |  | |
| **5. Artesunate/napthoquine** | | | Artemisinin | | Napthoquine | Artemisinin | Napthoquine | |
| 304/14 | Arco® tablets | | 102.3 | | 109.5 | 98.1 | 97.8 | |
| **6. Artesunate** | | | Artesunate | | - | - | - | |
| 312/14 | Artesun® injection | | 99.3 | | - | - | - | |

**Table S3 Results of microbial load carried out on the liquid and herbal anti-malarials**

One quinine suspension had contamination with 50 colony forming units of aerobic bacteria per ml.

| **Daru No.** | **Brand name** | **Active pharmaceutical ingredients** | **Microbial load** | |
| --- | --- | --- | --- | --- |
|  |  |  | **Total aerobic count (cfu^[[2]](#footnote-2)^ml)** | **Yeasts and moulds (cfu/ml)** |
| 297/14 | Malacur® suspension | Dihydroartemisinin/Piperaquine Phosphate | Nil | Nil |
| 292/14 | Lonart® suspension | Artemether/lumefantrine | Nil | Nil |
| 304/14 | Quinine mixture | Quinine | Nil | Nil |
| 305/14 | Topquine® suspension | Quinine dihydrochloride | 50 | Nil |
| 306/14 | Topquine® suspension | Quinine dihydrochloride | Nil | Nil |
| 307/14 | Quinine suspension | Quinine | Nil | Nil |
| 308/14 | Quinine suspension | Quinine | Nil | Nil |
| 309/14 | Nelquine® drops | Quinine dihydrochloride | Nil | Nil |
| 310/14 | Falciquin® suspension | Amodiaquine HCl | Nil | Nil |
| 311/14 | Leoquin® suspension | Chloroquine HCl | Nil | Nil |
| 313/14 | Remox®^[[3]](#footnote-3)^ solution | *Ajuga remota* | Nil | Nil |
| 314/14 | Remox® capsules | *Ajuga remota* | Nil | Nil |

1. [↑](#footnote-ref-1)
2. Cfu= colony forming units [↑](#footnote-ref-2)
3. White *Ajuga remota* extract in natural vegetable oil [↑](#footnote-ref-3)
